# Factors Associated with Failure of Pneumatic Reduction in Children with Ileocolic Intussusception

**DOI:** 10.3390/children8020136

**Published:** 2021-02-12

**Authors:** Alaa Younes, Sanghoon Lee, Jong-In Lee, Jeong-Meen Seo, Soo-Min Jung

**Affiliations:** 1 Department of Surgery, Samsung Medical Center, Sungkyunkwan University School of Medicine, Seoul 06354, Korea; dr_alaa111@hotmail.com (A.Y.); 4hooni@gmail.com (S.L.); jm0815.seo@samsung.com (J.-M.S.); 2 Department of Surgery, CHA Medical Center, CHA University School of Medicine, Seongnam 13496, Korea; gslji@hanmail.net

**Keywords:** ileocolic intussusception, pneumatic reduction, risk factors

## Abstract

Intussusception is one of the most common causes of intestinal obstruction in children. Pneumatic reduction is the treatment of choice and has a high success rate. The most common cause of pneumatic reduction failure is the presence of a pathological leading point. We aimed to identify other factors that can lead to pneumatic reduction failure in children with ileocolic intussusception. This was a retrospective study conducted in two centers. Data were collected from January 2013 to December 2014. A total of 156 patients were diagnosed with intussusception and underwent pneumatic reduction, with the exception of one patient with peritonitis. We included patients with ileocolic-type intussusception without apparent pathological leading points. Logistic regression analysis of clinical parameters was performed to identify factors associated with pneumatic reduction failure. Of 156 patients diagnosed with intussusception in both hospitals, 145 were enrolled in the study. The overall efficacy of pneumatic reduction was 85.7%, and surgical reduction was performed in 21 patients. Univariate analysis showed that a high segmented neutrophil count, low hemoglobin level, high body temperature, and higher weight percentile were associated with pneumatic reduction failure. Multivariate analysis showed that a high segmented neutrophil count, low hemoglobin level, and higher weight percentile were significantly associated with pneumatic reduction failure. Pneumatic reduction is safe and effective as a first-line treatment for pediatric intussusception. However, a high segmented neutrophil count, low hemoglobin level, and higher weight percentile are associated with the failure of this treatment.

## 1. Introduction

Intussusception is one of the most common causes of intestinal obstruction in children [1,2]. The diagnosis is usually confirmed by ultrasound, which has 98–100% sensitivity and 88% specificity [3]. Pneumatic reduction (PR) is the gold standard of treatment in children with intussusception and has been shown to be safe and effective, with reported success rates of over 80% [4,5,6]. However, when multiple attempts at PR fails to reduce intussusception, it must be performed manually by surgical means. Known contraindications for PR are peritonitis and intestinal perforation [7,8,9]. In addition, the presence of a pathological leading point (PLP) is a major factor associated with PR failure [10]. However, in a considerable number of cases without an apparent PLP, PR is unsuccessful in reducing intussusception, and surgical reduction is required. Here, we evaluated the factors associated with PR failure in pediatric cases of ileocolic intussusception without PLPs.

## 2. Materials and Methods

Data were collected retrospectively from two tertiary hospitals. All children aged 0–14 years who were diagnosed with intussusception between January 2013 and December 2014 were evaluated. The diagnosis and classification of intussusception were confirmed by ultrasound. Only patients with ileocolic-type intussusception were included, and patients with other types of intussusception and those with pathologically confirmed PLPs were excluded. All PR procedures were performed under fluoroscopy, and when PR failed, the patient was transferred to the operative room for surgical reduction.

Data on patient characteristics (age, weight, and weight percentile) and clinical and laboratory data (body temperature, white blood cell count with differential, hemoglobin levels, C-reactive protein levels, and absolute neutrophil count) were collected, and their association with PR failure was analyzed using univariate and multivariate logistic regression analyses. A receiver operating characteristic (ROC) curve was constructed to determine the optimum cutoff values of the significant factors. Statistical analyses were conducted using SPSS software (version 8.0, SPSS Inc., Chicago, IL, USA).

This study was approved by the Institutional Review Board of Samsung Medical Center (IRB number 2018-10-075), and the requirement for informed consent was waived.

## 3. Results

**Figure 1:**
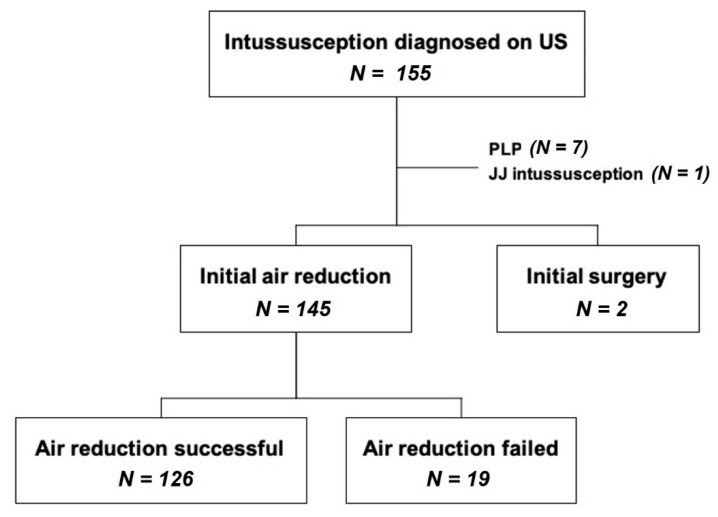
**Flowchart of patient selection** (PLP: pathological leading point, JJ: jejunojejunal).

In the two hospitals, 155 patients were diagnosed with intussusception during the study period. Ten patients were excluded from the study for the following reasons: two patients presented with signs of peritonitis and underwent surgery without PR, seven patients demonstrated PLPs on postoperative pathologic evaluation, and one patient had jejunojejunal intussusception. The remaining 145 children with ileocolic intussusception were analyzed (Figure 1).

The patient characteristics are shown in Table 1. The mean age at diagnosis was 24.6 months, and there were 95 boys and 50 girls. The body weight percentiles of approximately 56% of the patients were in the range of 25–75%.

Univariate analysis showed that a high segmented neutrophil count (*p* = 0.017, odds ratio [OR] 1.046, 95% confidence interval [CI] 1.008~1.086), low hemoglobin level (*p* = 0.01, OR 0.545, 95% CI 0.334~0.863), high body temperature (*p* = 0.049, OR 2.203, 95% CI 1.004~4.837), and high weight percentile (*p* = 0.015, OR 1.567, 95% CI 1.093~2.247) were significant factors associated with the failure of PR. Multivariate analysis was performed with the variables that were significant in univariate analysis and showed that a high segmented neutrophil count (*p* = 0.014, OR 1.052, 95% CI 1.010~1.095), low hemoglobin level (*p* = 0.018, OR 0.522, 95% CI 0.304~0.896), and higher weight percentile (*p* = 0.01, OR 1.668, 95% CI 1.032~2.457) were independent risk factors for PR failure (Table 2).

ROC curves were used to determine significant cutoff values associated with a negative impact on PR success. A segmental neutrophil level >67.3% (95% CI 1.735–15.609), hemoglobin level <12.2 g/dL (95% CI 2.247–22.86), and weight percentile >75% (95% CI 1.275–12.28) significantly affected the efficacy of PR.

## 4. Discussion

Intussusception is a common cause of acute abdomen in children requiring surgery. Fortunately, most patients can be treated with non-surgical treatments such as air enema reduction. PR is the first-line treatment for intussusception and is both safe and effective, with a reported success rate of more than 80% [4,11,12]. Surgical treatment is required for patients in whom PR fails after multiple attempts. In this study, PR failed in 12.9% of patients who subsequently underwent surgical reduction. Additionally, we identified three independent risk factors associated with pneumatic reduction failure in the absence of a PLP: low hemoglobin level, high segmental neutrophil level, and higher weight percentiles.

Early identification of patients who are likely to show PR failure is important for avoiding excessive PR. Intestinal perforation may occur if PR is performed with excessive air pressure in patients with a high probability of failure. Second, if pediatric surgeons receive an early notification regarding patients with a high probability of failure of air enema reduction, it is possible for them to be prepared to proceed with rapid surgical treatment after PR failure. Therefore, it is important to investigate objective indicators that can identify patients with a high probability of PR failure.

A symptom duration of >24 h, rectal bleeding, extremes of age, rectal prolapse, severe dehydration, and radiological signs of obstruction have been reported to negatively impact pneumatic reduction [5,12,13,14]. However, these factors are not considered absolute contraindications for PR. In this study, three factors negatively affected the effectiveness of PR: a low hemoglobin level (g/dL), high segmental neutrophil level (%), and high body weight percentile (%). A low hemoglobin level, which could be explained by rectal bleeding, has been reported to be significantly associated with PR failure [15], and a low hemoglobin level was significant when the level was <12.2 g/dL in the current study. Segmental neutrophils are also associated with PR failure [12], and in the current study, this parameter was significant if the level was >67.3%. However, the body weight percentile has not been addressed in previous reports, but it was associated with PR failure when the percentile was >75% in our series. Despite the presence of factors that affect its efficacy, PR remains the first-line treatment for pediatric intussusception in the absence of peritonitis and reduces the frequency of unnecessary laparotomy [12,16]. Our findings regarding the factors that negatively affect successful PR can be used to alert physicians to initiate an early surgical consultation.

This study had several limitations. First, this was a retrospective study, which is associated with risk of various bias in data collection and interpretation. Second, duration of symptoms, which is a well-known risk factor for failure of PR, was not included in the analysis [4,12,14]. Although exclusion of this variable was intended to minimize the possibility of recall bias, excluding symptom duration from the risk factor analysis may have impacted the results. Symptom duration is related to the variables found to be significant risk factors in this study. Longer duration of symptoms would lead to inflammation and venous bleeding from the bowel segment and subsequently elevated segmented neutrophil levels and decreased hemoglobin. Third, it is not possible to definitely confirm the absence of PLPs unless surgery is performed in these patients. Thus, a small proportion of patients among those who underwent successful pneumatic reduction actually had underlying PLPs. However, a patient with a PLP would be more likely to have recurrence of intussusception. Non-recurrence after pneumatic reduction in these patients supports the likelihood that they have been without PLPs at the time of successful pneumatic reduction.

In conclusion, although PR is highly effective in reducing intussusception in children, a low hemoglobin level, a high segmental neutrophil level, and higher weight percentiles were associated with pneumatic reduction failure in the absence of a PLP.

## Figures and Tables

**Table 1:** Patient demographics.

	*N* = 145 (%)
Age (months, mean ± S.D.)	24.6 ± 15.2
Boys:Girls	95:50
Body weight percentile, n (%)	
<3%	3 (2.1)
3~10%	13 (8.9)
10~25%	19 (13.1)
25~50%	46 (31.7)
50~75%	37 (25.5)
75~90%	17 (11.7)
90~97%	10 (6.9)
Body temperature (°C, mean ± S.D.)	36.9 ± 15.0
WBC (/uL, mean ± S.D.)	12,249 ± 6175
Segmented neutrophil (%, mean ± S.D.)	59.3 ± 17.6
Hemoglobin (g/dL, mean ± S.D.)	12.2 ± 1.7
C-reactive protein (mg/dL, mean ± S.D.)	0.7 ± 0.9

S.D. = standard deviation; WBC = white blood cell.

**Table 2:** Logistic regression analysis of clinical parameters for pneumatic reduction failure.

Variables	Univariate Analysis			Multivariate Analysis		
	*p* Value	OR	95% CI	*p* Value	OR	95% CI
Segmented neutrophil (%)	0.017	1.046	1.008~1.086	0.014	1.052	1.010~1.095
Temperature (°C)	0.049	2.203	1.004~4.837	0.158	1.997	0.765~5.210
Weight percentile (%)	0.015	1.567	1.093~2.247	0.010	1.668	1.032~2.457
Hemoglobin (g/dL)	0.010	0.545	0.334~0.863	0.018	0.522	0.304~0.896
Age (months)	0.078	0.969	0.935~1.004	-	-	-
Absolute body weight (kg)	0.226	0.901	0.761~1.067	-	-	-
C-reactive protein (mg/dL)	0.265	1.270	0.834~1.933	-	-	-
White blood cell (/uL)	0.441	1.000	1.000~1.000	-	-	-
Absolute neutrophil count (/uL)	0.382	1.000	1.000~1.000	-	-	-

## Data Availability

The data presented in this study are available on request from the corresponding author.

## Abbreviations

PR	pneumatic reduction
PLP	pathological leading point
